# The epigenetic circle: feedback loops in the maintenance of cellular memory

**DOI:** 10.1186/s13072-025-00621-6

**Published:** 2025-08-20

**Authors:** Marko Tomljanović, Cita Hanif Muflihah, Dejan Rajkovski, Pawel Mikulski

**Affiliations:** 1 International Institute of Molecular Mechanisms and Machines, Polish Academy of Sciences, Warsaw, Poland; 2 Nencki Institute for Experimental Biology, Polish Academy of Sciences, Warsaw, Poland

## Abstract

The memory of gene expression states, active or repressive, is a fundamental biological concept as it controls cell fate in development, immunity and abiotic stress responses. Such memory is maintained through cell division as a cornerstone of epigenetics. Cell division poses a threat to the stability of epigenetic memory as memory-encoding factors become diluted between daughter cells. Thus, long-term epigenetic memory must depend on the feedback loops to sustain it over cell generations.

Despite a widespread presence and fundamental importance, maintenance mechanisms of epigenetic memory are far from being clear. Here, we summarize present knowledge about feedback loops that allow maintenance of epigenetic information. We describe conceptually distinct, cis- and trans-, feedback loops, which rely on local, read-write propagation mechanisms or regulatory loops of diffusible factors, respectively. Furthermore, we provide cases of their frequent coupling in epigenetic systems in cells and synthesize current challenges in understanding feedback mechanisms. Overall, we believe this review to benefit the scientific community in bringing a holistic perspective on such fundamental biological phenomenon.

## Transcriptional output of stimulus-induced epigenetic memory

Epigenetic memory refers to the stable transmission of gene expression patterns from one cell generation to the next without changes to the underlying DNA sequence [1]. It constitutes a broad mechanism of the dynamic interaction between cells and their environment as it can be triggered or erased in stimulus-dependent manner. Epigenetic memory offers an opportunity for progeny cells to adjust their gene expression programs based on the stimulus received in their former generations. Such hysteretic behaviour can lead to differential transcriptional response when cells are exposed to recurrent stimuli in a phenomenon termed ‘priming’ or ‘acclimation’ [2]. It also allows a stable change of cell fate, exemplified by lineage differentiation in development [3].

**Fig. 1:**
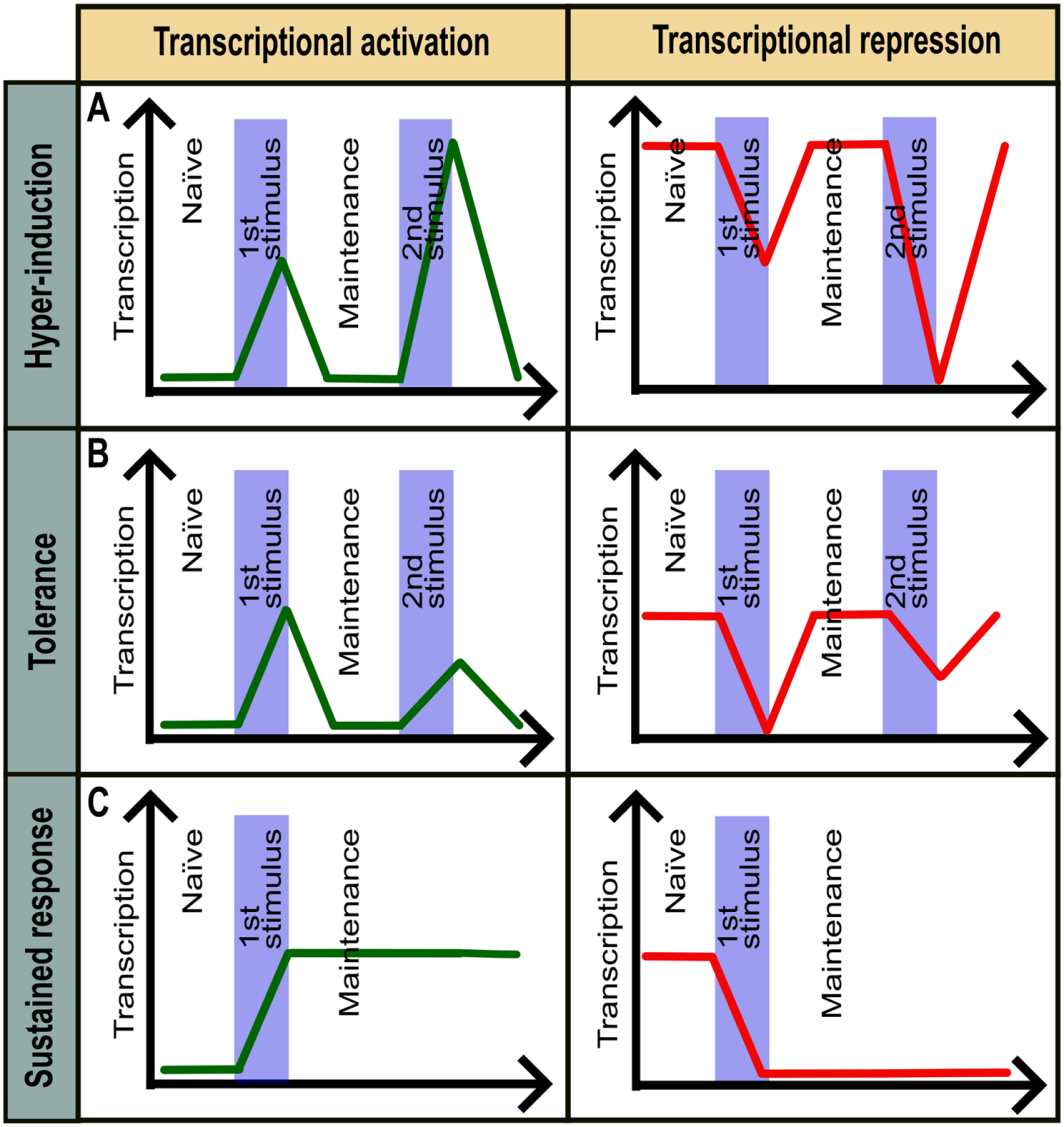
Common types of transcriptional output in stimulus-induced epigenetic memory. (**A**) Hyper-induction of stimulus-dependent transcriptional activation (left) or repression (right). (**B**) Tolerance (refractory response) in stimulus-dependent transcriptional activation (left) or repression (right). (**C**) Sustained transcriptional activation (left) or (repression) post-stimulus

Transcriptional consequences of stimulus-induced epigenetic memory are exemplified by the: (a) tolerance; (b) hyper-induction and (c) sustained response (Fig. 1). Tolerance corresponds to the cases where cells become more refractory to recurrent stimulations, leading to weaker transcriptional response after priming. For instance, in macrophages, repeated exposure to lipopolysaccharide (LPS) leads to reduced induction of pro-inflammatory genes such as *TNF* and *IL6*, a phenomenon associated with gradual chromatin closing and reduced recruitment of transcription factors [4]. In turn, hyper-induction corresponds to increased sensitivity to recurrent stimulations and augmented transcriptional responses after priming. Hyper-induction is exemplified by the interferon gamma-induced transcriptional memory allowing faster and stronger transcriptional activation of *GBP* family genes under recurrent stimulation [5–7]. Lastly, sustained response describes stable fixation of gene expression state induced by the stimulus. It is particularly observed in lineage differentiation in development. For example, during hematopoiesis, stimulus-driven expression of lineage-determining transcription factors (TFs) like PU.1 or GATA1 establishes stable, heritable gene expression profiles necessary for myeloid or erythroid identity [8, 9].

**Fig. 2:**
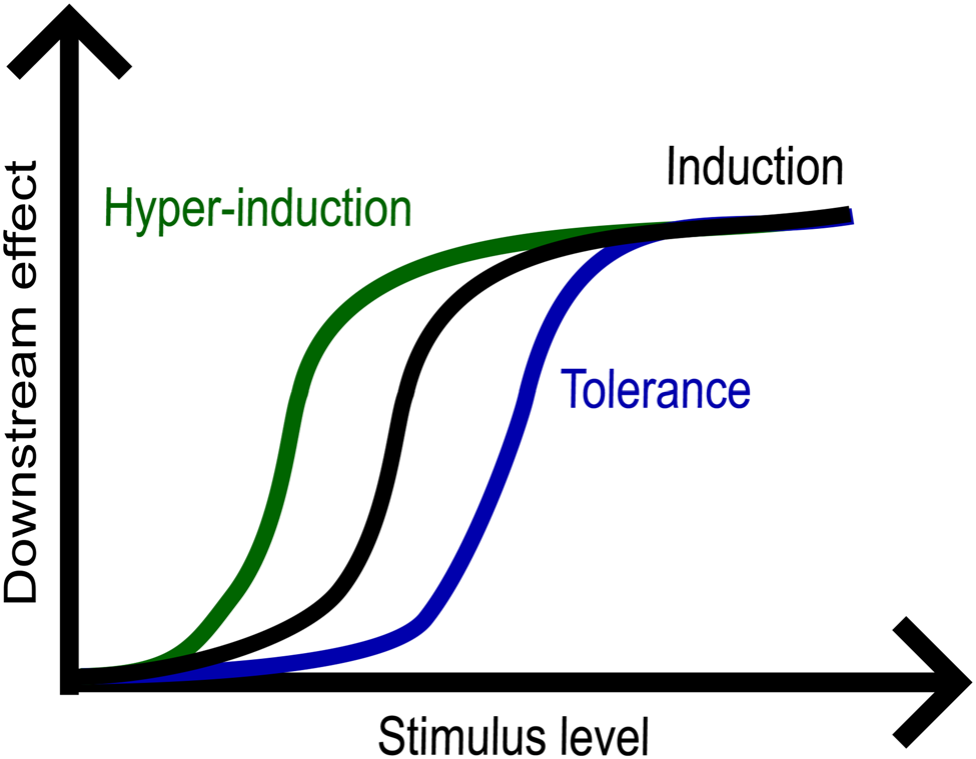
Conceptual representation of signalling activation energy in primed cells. Primed cells exhibit higher sensitivity (hyper-induction) or higher resistance (tolerance) to subsequent stimuli after initial priming event (induction). The responses lead to downstream effects– here: non-linear transcriptional activation

Stimulus-driven tolerance and hyper-induction are associated with the concept of signalling activation energy, analogous to chemical reactions (Fig. 2) [10]. It refers to a minimal level of stimulus (e.g. concentration) to trigger downstream events [10]. Mechanistically, once cells are primed by an initial stimulus, the signalling threshold required for subsequent changes under recurrent stimulation is altered. The threshold is increased in tolerance or decreased in hyper-induction cases to make cells more refractory or sensitive to stimuli, respectively. The examples of changed thresholds are well seen in immunological memory in T cells [10] and cell differentiation [11], elaborated below.

## Epigenetic memory phases

In stimulus-response context, the dynamics of epigenetic memory can be separated into establishment and maintenance phases. These phases, albeit extensively interconnected, are also controlled by partially distinct mechanisms. The maintenance of epigenetic memory can occur in the absence of initial stimulus and engage different regulatory factors than in establishment [12–14]. The research in various experimental systems allowed formulation of the ‘hit-and-run’ model, where memory establishment factors trigger changes at the target chromatin which are epigenetically maintained by distinct mechanisms. For instance, heat stress memory in Arabidopsis is established by a transient association of heat shock protein HSFA2, yet maintained by stable accumulation of H3K4me2 and H3K4me3 on their chromatin [15]. Similarly, immunological memory in T cells is established through opening of DNAse Hypersensitive Sites (DHSs) by inducible transcription factors (TFs) NFAT and AP-1, and maintained by constitutively expressed TFs - RUNX1 and ETS [14]. A comparable model has been also described for inflammatory memory in keratinocytes, where stimulus-specific TFs STAT3 establishes the memory and recruits FOS-JUN TFs to target loci. Subsequently, JUN presence is retained and allows secondary recruitment of the other epidermal homeostatic TFs, such as ATF3 and p63. While STAT3 and FOS actions are stimulus-induced and transient, the occupancy of homeostatic TFs at target loci is sustained post-stimulus and allows the maintenance of inflammatory memory [12]. In differentiation, hit-and-run model is exemplified by haematopoiesis. Here, transcription factor RUNX1 opens chromatin and activates expression of hematopoietic master regulators (e.g. PU.1) in hematopoietic precursor cells [16]. Subsequently, open chromatin sites are maintained by regulatory network involving PU.1 in the absence of RUNX1 and driving myelopoiesis [16]. Noteworthy, hit-and-run mechanisms have been also utilized in CRISPR-based epigenome editing [17].

## Feedback loops in the mechanisms of epigenetic memory maintenance

**Fig. 3:**
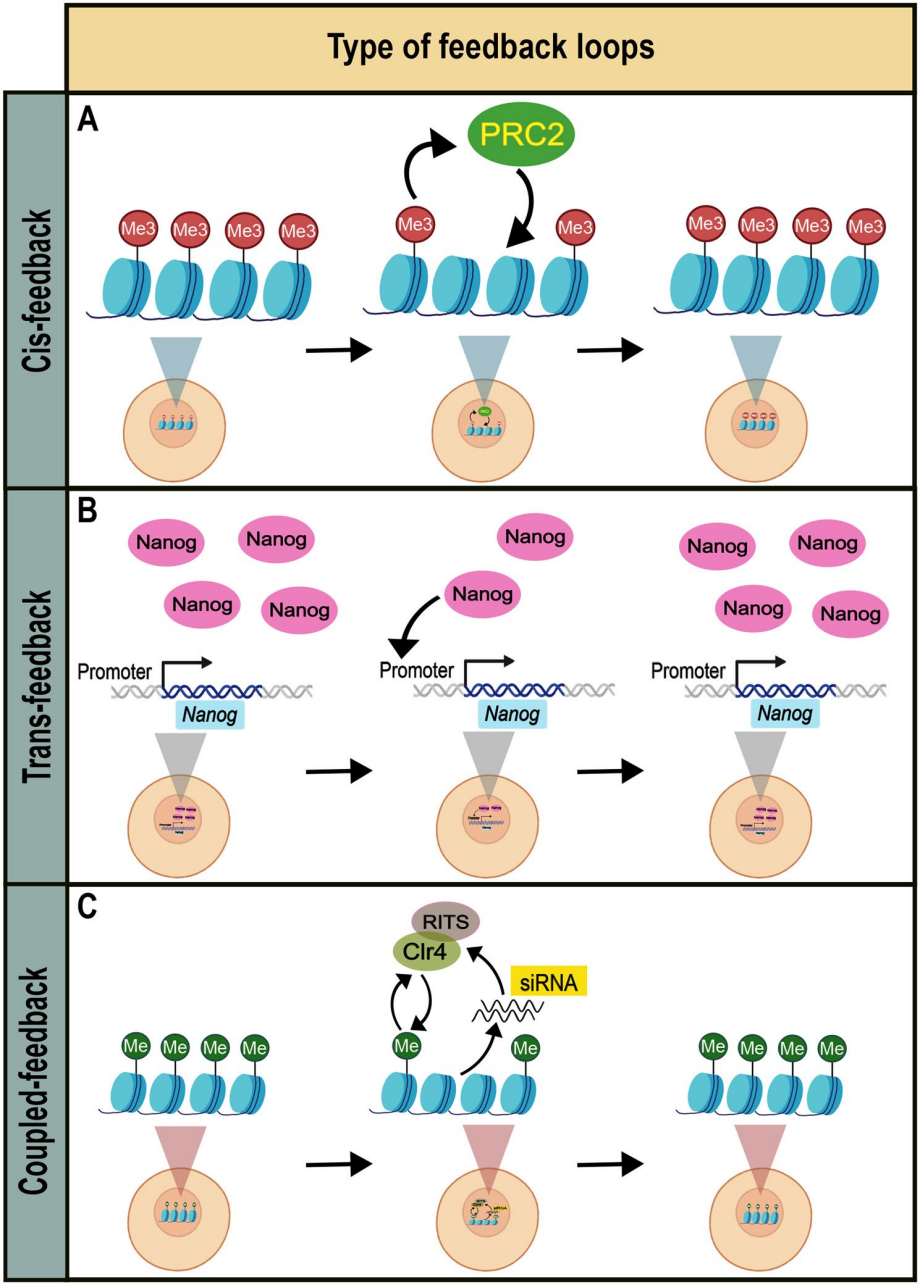
Examples of feedback loop types in the maintenance of epigenetic memory. (**A**) Cis-feedback. Read-write capability of Polycomb-repressive complex 2 (PRC2) allows re-establishment of H3K27me3 domain on target loci [32]. (**B**) Nanog TF binds to the promoter of its own gene and activates its expression forming an auto-regulatory feedback loop [71]. (**C**) Coupled-feedback. Interplay between cis-feedback through read-write capability of Clr4 and trans-feedback of siRNAs allows re-establishment of repressive H3K9me3 domain on target loci [92]

The mechanisms behind epigenetic memory phases, mentioned above, have been a subject of intensive research over the last decades. The maintenance of epigenetic memory is particularly interesting as it poses a challenge to preserve it over cell division such as mitosis. Faithful maintenance of epigenetic memory needs to withstand dilution and segregation of diffusible factors and chromatin components into daughter cells, as well as structural rearrangements such as global condensation of mitotic chromosomes. Therefore, the maintenance of epigenetic memory requires extensive, self-propagating feedback loops to sustain its effects over mitosis (Fig. 3) [18, 19].

Feedback loops required for memory maintenance can be categorized into cis- and trans-types. Cis-feedback loops correspond to cases where the carriers of epigenetic information are physically associated with the molecular entity they regulate such as chromatin [18]. Its predominant examples comprise read-write self-propagation of selected DNA and histone modifications at local genomic regions [18]. In turn, the maintenance by trans-feedback loops refer to the mechanisms where epigenetic information is stored in the concentration of diffusible factors, spatially separated from the entity they regulate [18]. Such trans-feedback loops rely on interplay of diffusible factors, exemplified by gene regulatory networks (GRNs) maintaining developmental cell fates [18]. Noteworthy, cis- and trans-feedback loops are frequently intertwined in cells, forming a coupled mechanisms [20]. In this review, we discuss the cases of such cis- and trans-feedback loops, as well as their coupling in epigenetic memory maintenance.

## Cis-feedback: read-write mechanisms in the maintenance of epigenetic memory

Cis-feedback loops in the maintenance of epigenetic memory entail read-write mechanism, where catalytic effectors (as “writers”) have the ability to recognize their own products (as “readers”) [18, 19]. Such recognition occurs either directly by the same factor or as a complex via associated non-catalytic subunits. Read-write mechanisms correspond mainly to, transcriptionally repressive or permissive, epigenetic chromatin modifications. In cell division, parental chromatin becomes diluted - mixed with newly synthesized DNA and histones, and segregated to daughter cells. Read-write mechanisms allow epigenetic memory to be maintained by recognition of residual enrichment of parental epigenetic modifications and its full restoration to neighbouring DNA region through positive, self-templating feedback loops.

### Repressive cis-feedback loops

The canonical example of repressive cis-feedback loops concerns 5-methylcytosine (5mC) DNA methylation. DNA methylation was the first discovered epigenetic modification, and, since its discovery, it has become arguably one of the most studied, being implicated in maintaining genome integrity, stable silencing of X chromosome, and developmental imprinting [21–23]. In mammals DNA methylation is catalyzed by three functional enzymes, DNA methyltransferase 1, 3 A and 3B (DNMT1, DNMT3A, DNMT3B), where DNMT1 is considered to be the primary ‘writer’ responsible for maintaining methylation patterns over cell division [24]. DNA methylation is propagated by re-establishment shortly after synthesis of a new DNA chain (DNA replication-coupled) or afterwards (DNA replication-uncoupled) [25]. DNMT1 interacts with partner proteins that are necessary for its localization and modulation of methyltransferase activity. Amongst them is E3 ubiquitin-protein ligase UHRF1. Through its SRA domain, UHRF1 binds to hemi-methylated DNA and recruits DNMT1 to re-establish methylation on the other DNA strand [26, 27]. Such mechanism constitutes a positive cis-feedback loop, in which epigenetic mark of DNA methylation on one strand directs the methylation of newly synthetized strand, thereby allowing the propagation of epigenetic signal to another generation of cells.

Methylation of H3K9 (H3K9me2/3) plays an important role in the formation of constitutive heterochromatin on, e.g. centromeres and telomeric regions. There are several enzymes responsible for the establishment of this repressive epigenetic mark in mammals, namely: SUV39H1, SUV39H2, SETDB1, SETDB2, G9A and GLP. Histone methyltransferase SUV39H1 contains a chromodomain on its N terminus that selectively binds to the H3K9me2/3 itself [28]. This provides the basis for a direct read-write mechanism in which a single enzyme is capable of establishing a mark and binding it to promote the propagation onto larger domains in the genome. In addition, H3K9me2/3 engages in another positive feedback loop involving a ‘reader’ - Heterochromatin-protein 1 (HP1). HP1 binds H3K9me2/3 through its chromodomain and recruits H3K9 methyltransferases SUV39H1/2 to establish propagation of H3K9me2/3 heterochromatin domains [29, 30]. This process is evolutionally conserved, present even in yeast *S. pombe*, where homologs of HP1 and SUV39H, Swi6 and Clr4, respectively, are involved in the establishment of H3K9 methylation at a single focal point and its spread to large chromosome domains [31].

Trimethylation of H3K27 (H3K27me3) is linked to gene repression and plays a crucial role in shutting off the transcription of genes that determine cell lineage decisions and pluripotency. H3K27me3 is established by Polycomb Repressive Complex 2 (PRC2), a multi-protein complex consisting of four essential components (EZH1/2, SUZ12, EED, RbAp46/48) and auxiliary subunits which ensure the appropriate spatiotemporal H3K27me3 deposition [32]. The subunit EED contains aromatic cage structure which allows it to bind to the mark H3K27me3 itself [33]. The binding causes allosteric activation of the PRC2 and deposition of H3K27me3 on surrounding nucleosomes thereby establishing a positive feedback loop that can extend for several dozen kilobases [34].

Aforementioned examples display cis-feedback loops in the propagation of individual chromatin modifications. However, there is a significant amount of crosstalk between various cis-feedback loops with the goal of creating heritable patterns of epigenetic memory. For instance, maintenance of heterochromatin involves interconnected feedback of DNA and H3K9 methylations, and histone deacetylation. In detail, HP1 ‘reader’ not only recruits H3K9 methyltransferases, but also DNMT1 and HDACs [35–37]. In turn, UHRF1 recognizes, not only a hemi-methylated DNA, but also H3K9me2/3 via its Tudor domain [38] and recruits HDAC1 [39]. Furthermore, both DNMT3 enzymes, in complex with DNMT3L, can recognize and bind unmethylated H3K4, which facilitates the establishment of new DNA methylation [40, 41]. Additionally, binding of DNMT3 enzymes to H3K36me2/3 serves as an anchor to tether them to specific genomic locations, such as gene bodies [42, 43].

An elegant study conducted in S. cerevisiae, which does not naturally possess methylated H3K9, has demonstrated that a faithful propagation of heterochromatin relies on a minimal dual cis-feedback loop of intertwined H3K9 methylation and H4K16 deacetylation. In this case, epigenetic maintenance was achieved by two synthetic chimeric proteins– H3K9 methyltransferase with recognition domain for deacetylated H4K16ac and H4K16ac deacetylase with recognition domain for methylated H3K9 [44].

Noteworthy, a crosstalk between feedback loops can be also antagonistic, exemplified by interplay between H3K9 methylation and H3S10 phosphorylation, one of the core marks of chromatin in dividing cells. The phosphorylation of H3S10, catalyzed by Aurora kinases, interferes with HP1 binding to H3K9 and causes its dissociation from chromatin [45, 46]. This is particularly interesting since the level of H3S10ph increases during cell division, thereby providing a mechanistic link between proliferative signals and changes in the chromatin [45].

### Transcriptionally permissive cis-feedback loops

In contrast to gene repression, read-write mechanisms in the maintenance of active transcriptional state are scarcely described and unclear. In fact, the inheritance of active expression state (‘transcriptional memory’) has been canonically believed to exclude self-templating mechanisms [47] due to artificial induction achieved formerly only by deleting endogenous regulatory elements [48, 49] or removing silencing effector proteins [50, 51]. However, read-write propagation has been recently shown for selected permissive chromatin modifications.

Di- or trimethylation of H3K36 (H3K36me2/3) is implicated in chromatin remodelling, transcription, DNA repair and mRNA splicing. In mammals, H3K36me2/3 is catalyzed by a broad range of enzymes, grouped into SETD2, NSD, ASHL, SETMAR and SMYD families [52]. The prime example of such ‘writers’ include NSD2, responsible for H3K36me2. NSD2 contains a PWWP domain that binds to H3K36me2, which forms the basis of a positive feedback loop that enables the establishment of this mark on large parts of the genome [53–55].

Dimethylation of H3K4 (H3K4me2) has been shown to control transcriptional memory of inosine metabolism in yeast [56, 57]. Read-write maintenance of H3K4me2 is conferred by a binding of Sfl1 to cis-regulatory element of *INO1* gene and anchoring it to nuclear periphery, followed by an establishment of H3K4me2 mark by modified COMPASS complex which lacks Spp subunit. In turn, deposited H3K4me2 is recognized by SET3C protein which recruits Spp- COMPASS complex, forming a feedback loop necessary for propagation of the memory onto the daughter cells [56, 57]. Similarly to H3K4me2, trimethylation of H3K4 (H3K4me3) in yeast is propagated by a COMPASS complex, containing Spp1 subunit. In this case, a read-write feedback is achieved by a recognition of H3K4me3 by Spp1 through its PHD domain and recruitment of COMPASS complex to re-establish methylation of newly incorporated histones after DNA replication [58, 59].

Compared to histone lysine methylation, which can have an activating or repressive effect on gene transcription, histone lysine acetylation is strongly correlated with transcription. One of the most characterized histone acetyltransferase (HAT) is p300/CBP complex. p300 interacts with more than 400 proteins, including many transcription factors, which makes it one of the central epigenetic hubs for regulating gene expression [60]. In vitro, p300 acetylates N terminal tails of all 4 histones, introducing multiple histone marks, such as: H3K9ac or H4K16ac [61]. p300 has a capability to bind its own catalytic products on a particular histone in bromodomain-dependent manner and propagate the acetylation onto other components of the nucleosome [61]. In this way, p300 ensures the preservation of acetylation status of nucleosomes after DNA replication.

Active chromatin also displays multiple intertwined cis-feedback loops to ensure its epigenetic propagation. For instance, multiple HATs (e.g. NuA3/4, SAGA, HBO1) are capable of recognizing H3 methylation marks through their Tudor or PHD domains. In turn, the catalytic function of H3 methyltransferases (e.g. MLL1, MLL4) is stimulated by pre-existing histone acetylation marks [62]. Overall, such interconnection of multiple cis-feedback loops allows to re-instate broad, multi-factorial chromatin states.

## Trans-feedback: loops and networks in the maintenance of epigenetic memory

In contrast to cis-feedback, the trans-feedback loops involve storage of epigenetic information in the concentration of diffusible factors– proteins or RNA [18]. Such mechanisms are well known for transcriptional regulators which can bind to and activate or repress expression of their own genes, closing a self-perpetuating loop [63]. These loops can be direct in a form of auto-regulatory mechanisms, where a target protein or RNA binds to and regulates its own gene. Alternatively, such loops can be indirect and be based on gene regulatory networks (GRNs), where a target protein or RNA binds to and controls expression of the regulators upstream to its own gene. In cell division, diffusible components are diluted and segregated to daughter cells. Trans-feedback loops enable a diluted population of a diffusible factor to reinstate its expression status in daughter cells and maintain epigenetic information.

### Repressive trans-feedback loops

Trans feedback loops are exemplified by the maintenance of silencing non-coding RNAs, such as small-interfering RNAs (siRNAs) and PIWI-interacting RNAs (piRNAs). As a common initial step, primary small RNAs (sRNAs) are generated through double-stranded RNA processing by Dicer. These primary sRNAs associate with Argonaute (AGO) proteins, forming an RNA-induced silencing complex (RISC) that targets complementary mRNAs for degradation or chromatin modifications [64, 65]. A key intermediate in sRNAs’ maintenance involves a synthesis of complementary RNA strands from targeted mRNAs by RNA-Dependent RNA Polymerases (RdRPs) [64]. Template and complementary RNAs form new dsRNAs which are processed by Dicer into secondary sRNAs. Unlike primary sRNAs, secondary sRNAs do not require the initial dsRNA trigger and can continue the gene silencing cycle even after cell division, allowing for self-reinforcing feedback loop [65, 66].

Repressive trans-feedback loops concerns also a GRN-based maintenance of transcriptional status and resulting cell fate. For example, reciprocal negative regulation of PAX5 and BLIMP1 transcription factors underlies B cell development [67]. PAX5 gene activation will lead to the repression of BLIMP1 to maintain identity of resting and activated B cells [67]. In turn, BLIMP1 switches off PAX5 to promote and lock in differentiation into plasma cells [67].

### Transcriptionally permissive trans-feedback loops

The classic paradigm of trans-feedback includes a regulation of phage Lambda’s lytic-lysogenic cycles mediated by a balance of cI and Cro protein concentrations [68]. cI protein maintains the lysogenic state by activating its own expression as a positive feedback loop, while repressing the genes required for lytic growth through binding to operator sites O_R_1/O_R_2 and blocking transcription of lytic genes [69, 70]. In turn, Cro represses cI expression and competes with CI through binding to operator site O_R_3, allowing lytic gene expression and lytic state [69, 70]. Such combination of positive and negative trans-feedback loops allows the maintenance of lytic or lysogenic growth.

Autoregulatory feedback loops are also crucial in determining cell fate in development. In response to a stimulus, cells modulate expression of developmental regulators and their gene regulatory networks (GRNs). By action of autoregulatory loops within these GRNs, cells maintain (self-renew) or change fate (differentiate) even in the absence of initial stimulus. One of the predominant examples of such loops comprises pluripotency factors, Oct4, Sox2 and Nanog. These TFs bind promoters of their own genes and across each other, as well as co-occupy several hundred downstream target genes [71]. Ultimately, such a combination of direct and indirect regulatory loops forms a circuitry to sustain expression of pluripotent TFs, leading to the maintenance of self-renewal capability [72].

Similar behaviour has been also shown for the master TFs of the other, healthy and diseased, cell types [73, 74], suggesting a generality of autoregulatory loops and GRNs. The Forkhead box A (FOXA) family, one of the most extensively studied pioneer master transcription factors (TFs), is known for its ability to bind target sites within closed chromatin, subsequently facilitating chromatin opening and controlling gene transcription across various cellular processes. FOXA2, in particular, has been reported to play a critical role in liver development and maintenance [75]. For instance, hepatocyte identity is maintained by a set of interconnected GRNs. These comprise a main regulatory circuit of hepatocyte-specific TFs, called Hep-ID, and an auxiliary non-hepatocyte-specific TFs network, termed Hep-IDCONNECT [74, 76]. The TFs of the Hep-ID network (e.g. HNF4A, FOXA2, NR1H4) form autoregulatory loops by binding to their own promoters and increasing their own expression. In turn, the expression of Hep-ID TFs reciprocally activates Hep-IDCONNECT TFs (e.g. NR1H3, THRB), forming a second positive feedback loop to preserve liver cell fate [74, 76]. Another example of an autoregulatory network is found in muscle differentiation among myogenesis TFs, involving MyoD, Myf5, and Myogenin. These factors activate each other’s transcription, forming a regulatory loop to maintain transcription of Myogenin, which preserves myoblast cell identity [77].

The maintenance of cell identity by autoregulatory loops and GRNs has been also described for immune cell types. The differentiation of T and B cells captures good examples of the preserved epigenetic memory, crucial for immune responses. For instance, upon the first activation of naive T cells, the recruitment of inducible TFs (NFAT and AP-1) creates chromatin opening at cis-regulatory elements called ‘primed DNase hypersensitivity sites’ or ‘latent enhancers’ [14]. Such changes enable binding of constitutively expressed TFs, RUNX1 and ETS-1, to previously inaccessible sites [14]. Recurrent binding of RUNX1 and ETS-1 signalling, maintains chromatin accessibility at primed elements after T cell activation ceases and during differentiation to dividing T-blast cells and quiescent memory T cells [14, 78]. Upon rechallenge, maintained active chromatin at primed elements allows stronger induction of nearby immune response genes. Noteworthy, the maintenance of a subset of primed elements in memory T cells requires subsequent reinforcement of inducible TFs (e.g. AP-1) by recurrent IL-2 and/or IL-7 signalling [79], closing a feedback loop.

Preserved epigenetic memory in B cells and macrophages depends on the optimal expression of master TF PU.1 and its coordination with cis-regulatory elements of its own locus. B cell development depends on moderate expression of PU.1, achieved by a feedback loop of PU.1 protein binding to -15/-14 kb upstream enhancer of its gene, co-occupied by B cell-specific TFs E2A/FOXO1 [80, 81]. In turn, macrophage identity relies on a high expression of PU.1, controlled by PU.1 binding of -12 kb upstream element, along with C/EBP family macrophage TFs [82]. In turn, PU.1, being a master GRN factor, occupies macrophage-specific cis-regulatory elements and activates latent enhancers to preserve macrophage identity and epigenetic memory to immune stimulation [83, 84].

## Coupled cis-trans feedback: when chromatin meets diffusible factors

Despite a reductionistic categorization, the cis- and trans-feedback loops in epigenetic memory maintenance do not exist as separate entities in cells but are rather substantially intertwined. The degree of such interconnectedness is frequently context-dependent, based on the other variables such as the strength of antagonistic feedback and proliferative potential of recipient cells [85, 86]. Conceptually, in a bistable model of transcriptional response, coupling of cis- and trans-feedback loops can determine stable fixation of opposing, active (ON) or repressed (OFF), gene expression state across cell generations or frequent, highly reversible switches between expression states (metastability).

**Fig. 4:**
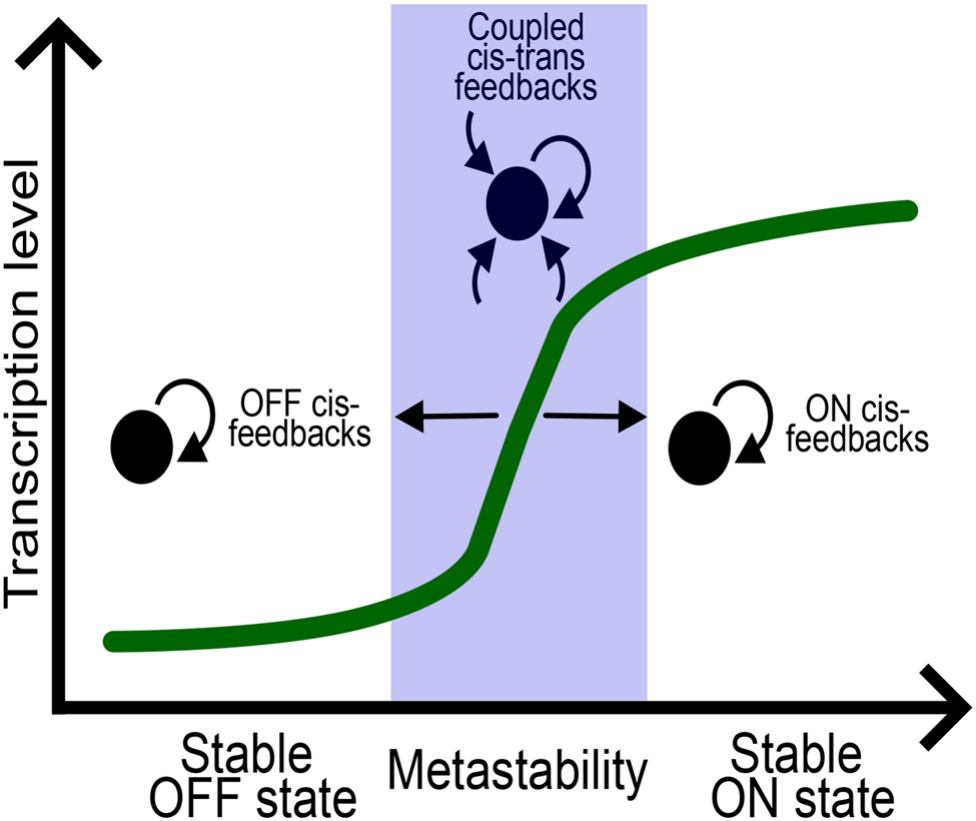
Interconnectedness of feedback types in example of Polycomb-mediated transcriptional regulation. Conceptual visualization of results from: [85– [90, 107]. The OFF state is stably maintained through read-write cis-feedback loop between H3K27me3-PRC2 as long as opposing, active transcription is low and Polycomb domain has sufficient number of nucleosomes [88, 89, 107]. Metastability (reversible switches between ON-OFF states) is achieved when repression and transcription are in equilibrium and Polycomb domain is short [85, 86]. In metastable state, Polycomb repression is maintained by coupled cis-trans feedback loops– H3K27me3-PRC2 read-write mechanism and action of PRC2-associated proteins [86]. When sufficiently strong transcriptional feedback occurs, Polycomb target genes switch to ON state [88, 90], capable of being maintained by cis-feedback [90]

In practical examples, mathematical modelling of Polycomb-mediated repression at *FLC* locus and its experimental validation on *FLC* transgenes [86–88]. These studies demonstrated that the OFF state is faithfully maintained over cell division by cis-feedback as long as the opposing feedback of active transcription is low [87, 88] and the locus contains sufficient number of Polycomb-modified nucleosomes [86] (Fig. 4). Consequently, short regions of repressive/active chromatin were predicted to be inherently metastable [89] and their mitotic maintenance require an additional trans-feedback from associated diffusible proteins [86] (Fig. 4). Furthermore, in mammalian neural progenitors, active expression state at Polycomb-target genes was shown to be epigenetically maintained by cis-feedback [90] and opposite, repressive state capable of being faithfully re-established by trans-feedback, independently of cis-acting, parental nucleosomes [90].

Fixation of stable, heritable states or their reversibility have crucial implications in cell fate determination in development and stress responses as exemplified by tumorigenesis induced epigenetically upon transient loss of Polycomb repression in Drosophila [91], a switch to generative development in vernalized plants [87] and stable activation of cell fate regulators upon transient Polycomb loss in mammalian cells [90].

Precise assessment of the reliance of epigenetic systems on either feedback type (or their intertwining) and their phenotypic consequences have been hard to prove experimentally. However, there are cases where the maintenance of epigenetic factors relies on coupled cis-trans feedback loops between chromatin and diffusible factors, as exemplified below.

### Repressive cis-trans feedback loops

The prime example of coupled cis-trans feedback loops forming a minimal machinery of epigenetic memory maintenance concerns a crosstalk between small RNAs and chromatin modifications. For instance, experiments conducted in *S. pombe* [92] have revealed a mechanism for transcriptional repressive network incorporating repressive mark H3K9me3 which has the potential for self-propagation, as described beforehand, and constitutes the cis- component of the feedback loop and siRNA capable of inducing heterochromatin on distant loci, thereby acting as trans- component of this network. By using transgene cen::ade6 + inserted near a centromere, which is repressed by heterochromatin, it has been demonstrated that the siRNA transcribed from the repressed locus can cause repression of endogenous ade6 + gene and other nearby genes. Such repression depends on the establishment of H3K9me3 mark over endogenous ade6 + and surrounding genes, where it is stably passed on to the progeny over up to 32 divisions. The maintenance of transcriptional repression depends on, both siRNA and H3K9me3, as indicated by perturbation experiments [92]. The siRNA-induced establishment of H3K9me3 has been also discovered in *C. elegans*, in which *smg-1* RNAi caused the formation of 7 kb H3K9me3 domain around smg-1 locus. This silencing is epigenetically maintained, in the absence of an initial RNAi signal, for up to two generations [93]. Similar crosstalk has been observed for piRNAs involved in transcriptional gene silencing (TGS) of transposon loci. piRNAs complexed with specific PIWI proteins translocate to the nucleus, bind nascent transcripts of target transposon loci and trigger their chromatin repression by recruiting H3K9 and DNA methyltransferases [94, 95]. In turn, a study on mice suggested that a single transcriptional activation of convergent GM-CSF transgene is sufficient to trigger formation of transient siRNAs. Which then mediate self-sustaining maintenance of repressive chromatin dependent on H3K9me3 and H3K9 hypoacetylation, but not DNA methylation [96].

Another example of coupled cis-trans feedback in epigenetic maintenance concerns DNA methylation. Despite having a potential for self-propagating system through minimal UHRF1-DNMT1 read-write mechanism, the maintenance of DNA methylation has been shown to rely also on posttranslational modifications on chromatin and replication factors. For instance, UHRF1 can also ubiquitinate diffusible factor - PAF15 (PCNA-associated factor 15), which binds DNMT1, promotes its recruitment to DNA during the S phase and increases the efficiency of DNA methylation re-establishment on daughter DNA strands [97]. Relevantly, in addition to a read-write mechanism, DNA methylation status of cis-regulatory elements and gene promoters has been observed to be maintained through the association between TFs and DNA methylation effectors, e.g. PPARγ-TET1 [98] or GCNF-DNMT3 [99] interactions. In turn, DNA methylation status affects local binding of TFs [100], closing a feedback loop.

Epigenetic maintenance based on cis-trans feedback loops has been also described in the interplay between long non-coding RNAs (lncRNAs) and chromatin modifiers. One of the main relevant, yet context-dependent, examples comes from X chromosome inactivation studies. LncRNA Xist establishes X chromosome silencing by recruiting co-repressors such as: RNA-binding proteins (e.g. SPEN, hnRNPK), chromatin-modifying complexes (e.g. PRC1, NCOR/SMRT) or chromatin structural factors (e.g. SMCHD1) [101, 102]. Xist has been long believed to be dispensable for inactive X maintenance in somatic cells, which is instead controlled by late repressive pathways (e.g. DNA methylation). However, more recent studies in B cells and hPSCs suggest the involvement of Xist in early maintenance phases and for X-encoded genes with lower CpG methylation levels at their promoters [103, 104]. Such observations suggest that epigenetic maintenance of X inactivation depends on the redundant pathways, forming cis- and trans-feedback loops, with varying degrees of contributions based on the cell-type and strength of antagonistic feedback loops.

Furthermore, coupled cis-trans feedback loops can be also observed in the maintenance of facultative, Polycomb-regulated and H3K27me3-marked, heterochromatin. PRC2 has an aforementioned capability to confer read-write mechanism and self-propagate H3K27me3 on the chromatin after cell division. However, in the context of strong antagonistic feedback of transcriptional activators and/or ongoing DNA replication, epigenetic maintenance of Polycomb heterochromatin requires, not only a read-write capability, but also a feedback from trans-acting DNA binding proteins [86, 87]. In particular, restoration of H3K27me3 enrichment in Drosophila requires a presence of intact DNA sequence, Polycomb Responsive Element (PRE), which nucleates H3K27me3 and allows its spreading [105, 106]. Importantly, such coupled mechanism is crucial specifically for H3K27me3 maintenance during ongoing replication, but dispensable in replication-stalled cells [105, 106]. A similar mechanism has been described also in vernalization paradigm in Arabidopsis, where long-term maintenance of FLC repression depends on DNA sequence and H3K27me3 occupancy at the nucleation region [86, 107]. Conversely, de novo establishment and maintenance of heritable H3K27me3-mediated gene repression in proliferating mammalian stem and progenitor cells after PRC2 inhibition involve also a feedback from repressive and activating trans-factors [90, 108].

### Transcriptionally permissive cis-trans feedback loops

A cooperation between cis- and trans-feedback loops can be also seen in the maintenance of active expression state. Such coupled feedback loops are exemplified by transcriptional memory maintained by an interplay between active chromatin modifications, transcription factors and transcription itself [109–112]. Initiated by the assembly of the preinitiation complex (PIC), the inherently complex nature of transcription underscores the even greater intricacy of the cis–trans regulatory feedback mechanisms involved [113]. In detail, active histone modifications facilitate recruitment of transcription factors, which allows binding of Polymerase II complexes and activation of transcription [109–112]. In turn, activated transcription and engaged Polymerase II promote a recruitment of chromatin modifiers and transcription factors to re-instate particular active histone modifications [109–112]. Similarly to a repressed expression state, such mechanism, in principle, allows the re-establishment of active chromatin after dilution caused by DNA replication. Transcription activation can happen either directly after DNA replication or throughout the cell cycle [114, 115]. The examples of such coupled feedback loops concern pathways centred around, e.g. H3K4me3, H3K4me1, H3K36me3 and H3/H4 pan-acetylation.

For instance, pre-existing H3K4me3 at target genes’ TSSs is directly recognized by H3K4me3 reader such as TAF3, a subunit of TFIID general transcription factor complex. TFIID binds to the target genes through TAF3, on the top of recognition of the TATA box through TBP subunit, and allows assembly of PolII pre-initiation complex (PIC) [116]. In turn, PIC recruits H3K4me3 methyltransferase complexes (COMPASS or COMPASS-like) to locally catalyze H3K4me3 at the target genes, allowing further re-establishment of TFIID binding, recruitment of PIC and gene transcription [116]. The recognition and restoration of H3K4me3 through transcriptional machinery have been described also for other H3K4me3 readers: chromodomain-containing chromatin remodellers (e.g. CHD1, NURF), Integrator and Mediator complexes, elongation factors (e.g. PAF, FACT) or Tudor domain-containing chromatin reader Spindlin 1 [59, 117]. Furthermore, an elegant model for epigenetic maintenance has been also proposed for H3K4me1 pathway. In Arabidopsis vernalization paradigm, FLC active transcriptional state before cold is maintained through a positive feedback loop between chromatin modifications, transcription and RNA processing. Co-transcriptionally deposited H3K4me1 promotes high PolII processivity and distal transcript termination [118]. In parallel, such effects inhibit actions of 3’ RNA processing machinery (e.g. CPF, FCA) which ultimately prevents recruitment of H3K4me1 demethylase FLD (LSD1 homologue), stimulating further transcription rounds and restoration of H3K4me1 in gene body to lock system in a stable feedback loop [118].

In turn, H3K36me3, marking actively transcribed gene bodies, has been shown to be recognized by transcriptional co-activators such as N-Pac [119]. N-Pac interacts with pTEFB and Ser2/Ser5-phosporylated PolII, promoting transcriptional elongation [119]. In turn, elongating PolII recruits methyltransferase SETD2 to co-transcriptionally re-deposit H3K36me3 and allow its recurrent recognition by co-activators [119]. Another example of coupled cis-trans feedback loops corresponds to histone acetylation pathways. Pan-acetylation at multiple histone residues (e.g. H3K27, H3K9, H4K12) is catalyzed co-transcriptionally by histone acetyltransferase complexes, such as SAGA or NuA4 [120, 121]. In turn, H3/H4 acetylation marks recruit bromodomain-containing reader proteins such as chromatin remodellers (e.g. BRD4 [122]), which facilitate recurrent transcription by promoting binding of PolII or transcription factors [120]. Noteworthy, the complexities of coupled cis-trans feedback are augmented by the crosstalk between various chromatin modifying pathways, exemplified by multi-domain reader proteins and complexes associated with transcriptional machinery (e.g. ZMYND family proteins [123] or Mediator complexes [124]).

An elegant case of epigenetic transcriptional memory comes also from cell fate regulatory networks [20]. For example, efficient establishment and stable maintenance of pluripotency in iPSCs has been demonstrated not only to rely on canonical pluripotency TFs (Oct4, Sox2, Klf4, Myc [125]), but also relieved activity of epigenetic factors such as NuRD nucleosome remodelling [126] and deacetylation complex, DOT1L H3K79 methyltransferase [127] or TET1 [128], a dioxygenase which plays a crucial role in removal of methylated cytosines from DNA [129].

## Mitotic stability of feedback loops

To reinstate gene expression states after mitosis, cis, trans and coupled feedback loops can act at various phases of cell cycle. High chromosome condensation [130] and disintegration of nuclear membrane in M phase [131] poses a particularly difficult challenge for feedback loops to maintain epigenetic information. The majority of transcription factors and chromatin modifiers become evicted from mitotic chromatin [132] and mitotic genome architecture, including Topologically Associated Domains (TADs) and A/B compartments, become largely dismantled [133]. In addition, given an extensive interplay between various factors, it is frequently unresolved which components of the feedback loops serve as primary factors that allow the maintenance of epigenetic information through M phase. However, the research over past decades has addressed such issues and given experimental proofs that specific factors remain associated with mitotic chromatin and allow preservation of the feedback loops to daughter cells.

In repressive feedback loops, a faithful preservation and enrichment on mitotic chromatin was proven for DNA and histone methylations in mammalian cells. Specifically, a comparison between interphase and mitotic chromatin showed a largely preserved abundance of H3K27me3 and H3K9me3 [134, 135]. In turn, CpG methylation is rapidly restored to 80–90% of initial level already within 30 min of replication [136]. Strikingly, mitotic chromosomes in embryonic stem cells (ESCs) are enriched, not only with these chromatin modifications, but also their associated read-write machinery. Specifically, an association with mitotic chromatin was observed for, e.g. H3K9 methyltransferases (Suv39h1 and h2) [137, 138] and reader (HP1) [139], PRC2 components, DNA methyltransferases (DNMT1 and 3) [137] and methyl-CpG-binding protein (Mecp2) [137]. Such results suggest that most of the chromatin machinery required to sustain H3K9me3, H3K27me3 and 5mC remain bound to ESC chromosomes during mitosis.

In transcriptionally permissive feedback loops, mitotic chromatin in ESC was shown to sustain levels of active histone marks - H3K4me1/2/3 and H3K36me3 [134, 135]. Binding to mitotic chromatin was also shown for H3K4me3 writer complexes, such as COMPASS-like complex with MLL/KMT2A, Menin, ASH2L and RbBP5 [140]. In addition, multiple other trans factors were observed to occupy mitotic chromatin in a phenomenon termed ‘mitotic bookmarking’. Mitotic bookmarking allows diffusible factors to preserve positional information in cis and sustain expression state of target genomic elements through mitosis. In ESCs, mitotic bookmarking was displayed for pluripotency TFs (e.g. SOX2 [141]), nuclear receptors (e.g. ESRRB [142]) and chromatin remodellers (e.g. SWI/SNF complex components [143]). In differentiated cell types, mitotic bookmarking concerns TFs such as RUNX1 in mammary epithelium [144], GATA2 in dermal fibroblasts [145]) and FOXA1 in hepatic cells [146]). Their retention in mitotic chromatin enables rapid, post-mitotic transcriptional reactivation of the correct target loci and, as a result, faithful preservation of cell identity. Furthermore, despite a global transcriptional downregulation, mitotic chromatin maintains residual, low level of transcription and local occupancy of transcriptional machinery [147, 148]. Upon mitotic exit, the amplitude of transcription becomes re-established [147]. Mitotic bookmarking and retention of permissive histone modifications on mitotic chromatin offers epigenetic maintenance of feedback loops for active gene expression state.

## Challenges and future research directions in epigenetic memory maintenance

The maintenance of epigenetic memory is critical for cell fate determination and stress responses. Although memory maintenance mechanisms have been a subject of research since decades, challenges and open questions still remain.

Firstly, the degree of interconnectedness between cis- and trans-feedback loops is still poorly characterized. The distinction between sufficiency and necessity of given feedback type in epigenetic systems frequently lacks experimental evidence. Such a gap is particularly important to describe molecular principles of bistability in epigenetic memory.

Secondly, recent developments in epigenetic mechanisms concerned more frequently gene silencing with much more confounded view on the memory of active transcription. With high instability of permissive chromatin modifications and more dynamic nucleosomal dispersion post-replication [149–151], it is particularly obscured which molecular entities allow a faithful passage of transcriptional memory to the next cell generation.

Thirdly, a comprehensive understanding of the mechanisms sustaining cis- and trans-feedback loops remains incomplete. For example, the minimal concentration of diffusible factors required to sustain trans-feedback loops across cell division is poorly defined. Conversely, the precise sequence of molecular events that enables faithful passage of epigenetic chromatin modifications during DNA replication is not yet sufficiently characterized.

Finally, the aberrant regulation of feedback loops in pathological state remains partially described. Development of diseases such as cancer involves mutations of epigenetic regulators and decreases in robustness in the maintenance of epigenetic information [152]. Relatedly, erosion of epigenetic information causes loss of cellular identity as one of the hallmarks in aging in mammalian cells [153], supported by the mechanistic studies in, e.g. yeast [154]. It can be envisaged that such deregulation involves perturbations of particular cis- and trans-feedback loops. However, functional studies linking infidelity in the maintenance of epigenetic memory to pathological state are still poorly characterized.

The resolution of above challenges can come from a rapidly developing state of technology in epigenetic research. The particularly beneficial advancements concern an enhancement of spatial and temporal resolution in epigenetic processes. For instance, the new iterations of super-resolution techniques have allowed precise identification of specific genomic targets bound by epigenetically maintained factors during cell division phases [155]. In turn, recent developments of temporal chromatin enrichment assays have improved enrichment measurements at short timescales [156]. Such advancement has been particularly useful in assessing the kinetics of chromatin restoration post-DNA replication and identification of primary epigenetic chromatin modifications.

Overall, epigenetic memory maintenance is still characterized by multiple open ties and unclarities. However, current developments and the advancing state of knowledge offer a rich foundation for future exciting discoveries in epigenetic research.

## Data Availability

No datasets were generated or analysed during the current study.

## Glossary

Epigenetic memory: the stable transmission of gene expression patterns from one cell generation to the next without changes to the underlying DNA sequences.
Hysteresis: the output of a given system that is determined not only by the current input or stimulus, but also by the system’s previous historic states.
Constitutive heterochromatin: permanently condensed and transcriptionally inactive chromatin localized primarily at high copy number tandem repeats and transposons.
Facultative heterochromatin: condensed and generally transcriptionally silent but with retained potential to transition into an open, transcriptionally active state upon stimulation.
Bistability: a feature of a system that can exist in two, mutually exclusive stable states. In gene expression, bistability refers to a phenomenon where a gene can adapt transcriptionally ON or OFF state.
Metastability: frequent and reversible fluctuation between system states, e.g. ON and OFF in gene expression models.
